# Supplementary material for: Identification of Freezing-Responsive microRNAs and Their Targets in Chinese Jujube by Small RNA and Degradome Sequencing
Source: Int J Mol Sci. 2026 Sep 4;27(17):7912. doi: 10.3390/ijms27177912 (PMC13566890; doi:10.3390/ijms27177912)
Supplement: Supplementary file 1 [file ijms-27-07912-s001.zip › Supplementary Material S2/novel_miR_78.pdf]

Provisional ID	:	novel_miR_78
Score total	:	4.3
Score for star read(s)	:	-1.3
Score for read counts	:	0
Score for mfe	:	4
Score for randfold	:	1.6
Score for cons. seed	:	
Total read count	:	2603
Mature read count	:	2592
Loop read count	:	0
Star read count	:	11

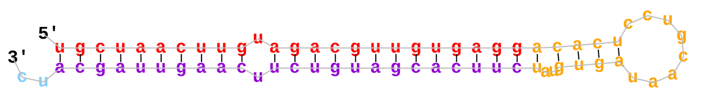

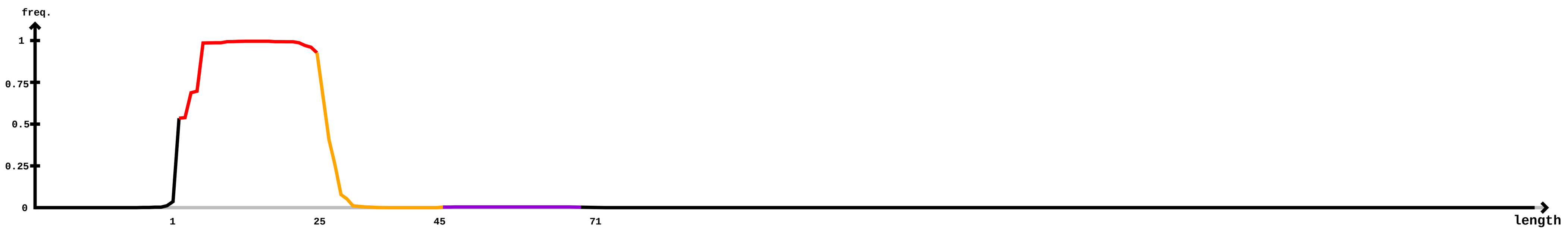

Mature Star

```
5'- ugcaguuuggcuuaggugagaaugcuaacuuguagacguugugaggacacuccugcaauaguguaucuucacgaugucuucaaguuagcauccucuuuugcgcaucauguauauauacauauauauaguuauauauauauauauauaugcacaucagucggugauugaugggcuguugcccaccaaaugguggggcgacuucauccauacuaguauucuuguauauacauuauguguguguguguguauaua  -3'  obs
    ugcaguuuggcuuaggugagaaugcuaacuuguagacguugugaggacacuccugcaauaguguaucuucacgaugucuucaaguuagcauccucuuuugcgcaucauguauauauacauauauauaguuauauauauauauauauaugcacaucagucggugauugaugggcuguugcccaccaaaugguggggcgacuucauccauacuaguauucuuguauauacauuauguguguguguguguauaua        exp
    ........((.((((.(((.((((((((((.(((((((((((((((((........)))).)))))))))))))).)))))))))))).))).)))).))....((((((((((((((((((((((((((...((((.((((..((((((((...)))))))).(..((((((((((....))).))))))).)...))))...))))...)))))))).)))))))))))))))))))))  reads  mm  sample
    ................ugagaaugcuaacuuguagacg...               1   0   JCK-1
    ................ugagaaugcuaacuuguagacg...               2   0   JCK-3
    ..................agaaugcuaacuuguagacg...               1   0   LT-1
    ..................agaaugcuaacuuguagacg...               2   0   LT-2
    ..................agaaugcuaacuuguagacg...               1   0   LT-3
    ....................aaugcuaacuuguagacguugu...           3   0   LT-3
    ....................aaugcuaacuuguagacguuguga...         2   0   LT-3
    ....................aaugcuaacuuguagacguuguga...         9   0   LCK-3
    ....................aaugcuaacuuguagacguuguga...         7   0   JCK-2
    ....................aaugcuaacuuguagacguuguga...         1   0   LT-2
    ....................aaugcuaacuuguagacguugugaA...        1   1   LT-1
    ....................aaugcuaacuuguagacguugugaggac...     1   0   LT-3
    .....................aGgcuaacuuguagacguugug...          1   1   LT-2
    .....................augcuaacuuguagacguugug...          1   0   JCK-1
    .....................augcuaacuuguagacguugug...          2   0   JCK-3
    .....................augcuaacuuguagacguugug...          3   0   LT-2
    .....................augcuaacuuguagacguugug...          2   0   JCK-2
    .....................augcuaacuuguagacguuguga...         2   0   JCK-3
    .....................augcuaacuuguagacguugugag...        17  0   JCK-3
    .....................augcuaacuuguagacguugugag...        7   0   JCK-1
    .....................augcuaacuuguagacguugugag...        7   0   LT-2
    .....................augcuaacuuguagacguugugag...        1   0   LT-3
    .....................augcuaacuuguagacguugugag...        16  0   JCK-2
    .....................Cugcuaacuuguagacguugugagg...       2   1   JCK-2
    .....................Cugcuaacuuguagacguugugagg...       4   1   JCK-3
    ......................ugcuaacuuguagacguu...             1   0   JCK-3
    ......................ugcuaacuuguagacguugu...           2   0   LT-2
    ......................ugcuaacuuguagaAguugu...           1   1   LT-3
    ......................ugcuaacuuguagacguugu...           1   0   LT-1
    ......................ugcuaacuuguagacguugu...           1   0   LT-3
    ......................ugcuaacuuguagacguugu...           2   0   JCK-1
    ......................ugcuaacuuguagacguugu...           4   0   LCK-3
    ......................ugcuaacuuguagacguugu...           1   0   JCK-3
```

Mature Star

ugcaguuuggcuuaggugagaaugcuaacuuguagacguugugaggacacuccugcaauaguguaucuucacgaugucuucaaguuagcauccucuuuugcgcaucauguauauauacauauauauaguuauauauauauauauaugcacaucagucggugauugaugggcuguugcccaccaaauggugggggcgacuucauccauacuaguauucuuguauauacauuauguguguguguauaua

......................ugcuaacuuguagacguuguU	2	1	LT-3
......................ugcuaacuuguagacguugug	3	0	JT-3
......................ugcuaacuuguagacguuguC	2	1	JCK-2
......................ugcuaacuuguagacguugug	3	0	JCK-3
......................ugcuaacuuguagacguugug	4	0	JT-2
......................ugcuaacuuguagacguugug	1	0	JCK-1
......................ugcuaacuuguagacguuguU	1	1	LT-1
......................ugcuaacuuguagacguugug	10	0	LCK-3
......................ugcuaacuuguagacguuguU	2	1	JCK-2
......................ugcuaacuuguagacguugug	2	0	JCK-2
......................ugcuaacuuguagaAguugug	1	1	JT-3
......................ugcuaacuuguagacguuguga	1	0	JCK-3
......................ugcuaacuuguagacguuguga	2	0	LT-3
......................ugcuaacuuguagacguuguga	2	0	JCK-2
......................ugcuaacuuguagacguugugag	1	0	LT-1
......................ugcuaacuuguagacguugugag	4	0	JCK-3
......................ugcuaacuuguagacguugugag	7	0	JCK-1
......................ugcuaacuuguagacguugugag	9	0	JCK-2
......................ugcuaacuuguagaAguugugag	1	1	JCK-2
......................ugcuaacuuguagacguugugagg	2	0	JT-3
......................ugcuaacuuguagacguugugagg	322	0	JCK-3
......................ugcuaacuuguagacguugugagg	26	0	LT-3
......................ugcuaacuuguagacguugugCgg	1	1	JCK-3
......................ugcuaacuuguagacguugugGgg	1	1	JCK-2
......................ugcuaacuuguagacguugugagg	219	0	JCK-1
......................ugcuaacuuguagacguugugagg	57	0	LT-1
......................uUcuaacuuguagacguugugagg	1	1	JCK-2
......................ugcuaacuuguagacguugugagA	1	1	JCK-3
......................ugcuaacuuguagacguugCgagg	1	1	JCK-1
......................ugcuaacuuguagacguugugagg	5	0	JT-2
......................ugcuaacuuguagaAguugugagg	2	1	LCK-3
......................ugcuaacuuguagaAguugugagg	4	1	JCK-3
......................uNcuaacuuguagacguugugagg	1	1	JCK-1
......................ugcuaacuuguNgacguugugagg	2	1	JCK-1
......................ugcuaacuuguCgacguugugagg	1	1	JCK-2
......................ugcuaacuuguagCcguugugagg	1	1	JCK-1
......................ugcuaacuuguagaAguugugagg	9	1	JCK-2
......................ugcuaacuuguagacguugugagg	45	0	LCK-3
......................Cgcuaacuuguagacguugugagg	1	1	JCK-2
......................ugcuaacuuguagaAguugugagg	2	1	JCK-1
......................ugcuaacuuguagacguugugagg	30	0	LT-2
......................ugcuaacuuguagacguuguNagg	1	1	JCK-1
......................ugcuaacuuguagacguugugagg	31	0	LCK-1
......................ugcuaacuuguagNcguugugagg	2	1	LCK-1
......................ugcuaaAuuguagacguugugagg	1	1	JCK-2
......................ugcuaacuuguagacguugugagg	30	0	LCK-2
......................ugcuaacuuguagacguugugagg	434	0	JCK-2
......................ugcuaacuuguagacguugugagga	1	0	JCK-1
......................ugcuaacuuguagacguugugagga	5	0	LCK-3
......................ugcuaacuuguagacguugugagga	2	0	JCK-2
......................ugcuaacuuguagacguugugagga	2	0	LT-2
......................ugcuaacuuguagacguugugaggaca	2	0	JCK-1
.......................gcuaacuuguagacguuguga	1	0	LT-3
.......................gcuaacuuguagacguugugagg	1	0	JCK-3
.......................gcuaacuuguagacguugugagg	3	0	JCK-2
.......................gcuaacuuguagacguugugagga	2	0	LT-1
.......................gcuaacuuguagacguugugagga	2	0	JCK-1
.......................gcuaacuuguagaAguugugagga	1	1	LT-1
........................cuaacuuguagacguuAu	1	1	LT-1
........................cuaacuuguagacguuguA	1	1	JCK-1
........................cuaacuuguagacguuguA	1	1	JCK-2
........................cuaacuuguagacguuguU	1	1	JCK-2
........................cuaacuuguagacguugugag	1	0	JCK-3
........................cuaacuuguagacguugugag	8	0	LCK-3
........................cuaacuuguagacguugugag	4	0	JCK-2
........................cuaacuuguagacguugugag	1	0	LT-1
........................cuaacuuguagacguugugag	1	0	LT-3
........................cuaacuuguagacguugugagg	8	0	JCK-1
........................cuaacuuguagacguugugagg	1	0	LT-1
........................cuaacuuguagacguugugagU	1	1	LT-2

```
                      Mature                Star
ugcaguuuggcuuaggugagaaugcuaacuuguagacguugugaggacacuccugcaauaguguaucuucacgaugucuucaaguuagcauccucuuuugcgcaucauguauauauacauauauauaguuauauauauauauauauaugcacaucagucggugauugaugggcuguugcccaccaaaugguggggcgacuucauccauacuaguauucuuguauauacauuaugugugugugguauaua
........................cuaacuuguagacguugugagU..........   1     1     JCK-1
........................cuaacuuguagacguugugagg..........   8     0     JCK-2
........................cuaacuuguagacguugugagg..........   8     0     JCK-3
........................cuaacuuguagacguugugagga.........   8     0     JCK-3
........................cuaacuuguagacguugugagga.........   2     0     LT-1
........................cuaacuuguagacguugugagga.........   19    0     JCK-1
........................cuaacuuguagacguugugagga.........   21    0     JCK-2
........................Uuaacuuguagacguugugaggac........   1     1     JCK-3
........................cuaacuuguagacguugugaggac........   83    0     JCK-3
........................cuaacuuguagacguugugaggac........   83    0     JCK-1
........................cuaacuuguagacguugugaggac........   1     0     LT-3
........................cuCacuuguagacguugugaggac........   1     1     JCK-3
........................cuaacuuguagacguugugaNgac........   1     1     JCK-1
........................cuaacuuguagNcguugugaggac........   1     1     JCK-1
........................cuaacuuguagacguugugaggac........   119   0     JCK-2
........................cuaacuuguagaAguugugaggac........   1     1     JCK-1
........................cuaacuuguagacguugugaggaca.......   2     0     JCK-1
........................cuaacuuAuagacguugugaggaca.......   1     1     JCK-3
........................cuaacuuguagacguugugaggaca.......   1     0     JCK-3
........................cuCacuuguagacguugugaggaca.......   1     1     JCK-1
........................cuaacuuguagacguugugaggaca.......   1     0     JCK-2
.........................uaacuuguagacguugugagga.........   4     0     JCK-1
.........................uaacuuguagacguugugagga.........   3     0     JCK-2
.........................uaacuuguagacguugugaggac........   2     0     LT-3
.........................uaacuuguagacguugugaggac........   1     0     JCK-2
.........................uaacuuguagacguugugaggac........   1     0     JCK-3
.........................uaacuuguagacguugugaggaca.......   2     0     JCK-2
.........................uaacuuguagacguugugaggaca.......   1     0     JCK-1
.........................uaacuuguagacguugugaggaca.......   2     0     JCK-3
.........................uaacuuguagacguugugaggacac......   1     0     JCK-2
.........................uaacuuguagacguugugaggacac......   1     0     JCK-1
.........................uaacuuguagacguugugaggacac......   1     0     JCK-3
.........................uaacuuguagacguugugaggacacu.....   1     0     JCK-3
.........................uaacuuguagacguugugaggacacu.....   4     0     JCK-2
..........................aacuuguagacguugugag...........   1     0     LT-1
..........................aacuuguagacguugugaU...........   2     1     LCK-3
..........................aacuuguagacguugugagg..........   1     0     JCK-3
..........................aacuuguagacguugugagg..........   9     0     LT-2
..........................aacuuguagacguugugagg..........   1     0     JCK-1
..........................aacuuguagacguugugagga.........   5     0     LT-1
..........................aacuuguagacguugugagga.........   3     0     JCK-2
..........................aacuuguagacguugugagga.........   2     0     LT-3
..........................aacuuguagacguugugagga.........   5     0     JCK-1
..........................aacuuguagacguugugagga.........   4     0     JCK-3
..........................aCcuuguagacguugugaggac........   1     1     LT-2
..........................aacuuguagacguugugaggac........   18    0     LT-2
..........................aacuuguagacguugugaggac........   32    0     JCK-2
..........................aacuuguagacguugugaggac........   12    0     JCK-1
..........................Cacuuguagacguugugaggac........   1     1     JT-1
..........................aacuuguagacguugugaggac........   4     0     LT-3
..........................aacuuguagacguugugaggac........   4     0     LT-1
..........................aacuuguagacguugugaggac........   14    0     JT-1
..........................aacuuguagacguugugaggac........   14    0     JCK-3
..........................aacuuguagacguugugaggaca.......   36    0     LT-1
..........................aacuuguagacguugugaggaca.......   43    0     LT-2
..........................aaNuuguagacguugugaggaca.......   1     1     LT-2
..........................Cacuuguagacguugugaggaca.......   1     1     JCK-2
..........................aCcuuguagacguugugaggaca.......   1     1     JCK-2
..........................aacuuguagacguugugaggaca.......   85    0     JCK-3
..........................aacuuguagacguugugaggaca.......   6     0     JT-3
..........................aacuuguagacguugugaggacC.......   1     1     JCK-3
..........................aacuuguagacguuAugaggaca.......   1     1     LT-2
..........................aacuuguagacguugugaggaca.......   7     0     JT-2
..........................aacuuguagacguugugaggaca.......   24    0     LT-3
..........................aacuuguagacguugugUggaca.......   1     1     JCK-1
..........................aacuuguagacguugugaggaca.......   56    0     LCK-3
..........................aCcuuguagacguugugaggaca.......   1     1     JCK-1
..........................aacuuguagacguugugUggaca.......   1     1     JCK-3
..........................aacuuguagacguugugaggaca.......   62    0     JCK-1
..........................aacuuguagacguugugaggaca.......   129   0     JCK-2
```

```
                   Mature                         Star
ugcaguuuggcuuaggugagaaugcuaacuuguagacguugugaggacacuccugcaauaguguaucuucacgaugucuucaaguuagcauccucuuuugcgcaucauguauauauacauauauauaguuauauauauauauauauaugcacaucagucggugauugaugggcuguugcccaccaaaugguggggcgacuucauccauacuaguauucuuguauauacauuauguguguguguauaua
..........................aacuuguagacguugugaggacac......................................................................................................................................................................................................   7    0    LT-1
..........................aacuuguagacguugugaggacac......................................................................................................................................................................................................   14   0    JCK-2
..........................aacuuguagacguugugaggacac......................................................................................................................................................................................................   21   0    LCK-3
..........................aacuuguagacguugugaggacac......................................................................................................................................................................................................   9    0    JCK-1
..........................aCcuuguagacguugugaggacac......................................................................................................................................................................................................   1    1    JCK-2
..........................aacuuguagacguugugaggacac......................................................................................................................................................................................................   6    0    LT-2
..........................aacuuguagacguugugaggacac......................................................................................................................................................................................................   4    0    LT-3
..........................aacuuguagacguugugaggacac......................................................................................................................................................................................................   4    0    JCK-3
..........................aacuuguagaAguugugaggacacu.....................................................................................................................................................................................................   1    1    LT-2
..........................aacuuguagacguugugaggacacu.....................................................................................................................................................................................................   9    0    LCK-3
..........................aacuuguagacguugugaggacacu.....................................................................................................................................................................................................   4    0    LT-3
..........................aacuuguagacguugugaggacacu.....................................................................................................................................................................................................   13   0    LT-2
..........................aCcuuguagacguugugaggacacu.....................................................................................................................................................................................................   1    1    LT-1
..........................aCcuuguagacguugugaggacacu.....................................................................................................................................................................................................   1    1    JCK-3
..........................aacuuguagacguugugaggacacu.....................................................................................................................................................................................................   25   0    JCK-2
..........................aacuuguagacguugugaggacacu.....................................................................................................................................................................................................   17   0    JCK-3
..........................aacuuguagacguugugaggacacu.....................................................................................................................................................................................................   1    0    LT-1
..........................aacuuguagacguugugaggacacu.....................................................................................................................................................................................................   29   0    JCK-1
..........................aacuuguagacguugugaggacacuU....................................................................................................................................................................................................   5    1    LCK-3
...........................acuuguagacguugugaggacacu.....................................................................................................................................................................................................   2    0    LT-3
............................cuuguagacguugugaggac........................................................................................................................................................................................................   2    0    LT-3
..............................uguagacguugugaggacacucc...................................................................................................................................................................................................   1    0    LT-3
..............................uguagacguugugaggacacucc...................................................................................................................................................................................................   4    0    LT-2
..............................uguagacguugugaggacacucc...................................................................................................................................................................................................   3    0    JCK-2
..............................uguagacguugugaggacacucc...................................................................................................................................................................................................   1    0    JCK-3
..............................uguagacguugugaggacacuccu..................................................................................................................................................................................................   1    0    JCK-1
..............................uguagacguugugaggacacuccu..................................................................................................................................................................................................   2    0    JCK-2
..............................uguagacguugugaggacacuccug.................................................................................................................................................................................................   1    0    JCK-2
..............................uguagacguugugaggacacuccug.................................................................................................................................................................................................   4    0    JCK-3
...............................guagacguugugaggacacuccugca...............................................................................................................................................................................................   1    0    JCK-2
................................uCgacguugugaggacacuc....................................................................................................................................................................................................   1    1    LT-2
................................uagacguugugaggacacuc....................................................................................................................................................................................................   3    0    LT-2
.................................agacguugugaggacacuccugc................................................................................................................................................................................................   2    0    LT-3
..................................................................cuucacgaugucuucaaguuag................................................................................................................................................................   1    0    JCK-2
..................................................................cuucacgaugucuucaaguuagc...............................................................................................................................................................   1    0    JCK-3
..................................................................cuucacgaugucuucaaguuagc...............................................................................................................................................................   1    0    JCK-1
..................................................................cuucacgaugucuucaaguuagca..............................................................................................................................................................   2    0    JCK-2
..................................................................cuucacgaugucuucaaguuagcU..............................................................................................................................................................   3    1    JCK-2
..................................................................cuucacgaugucuucaaguuagcU..............................................................................................................................................................   1    1    JCK-1
...................................................................ucacgaugucuucaaguuag.................................................................................................................................................................   1    0    JCK-1
...................................................................ucacgaugucuucaaguuagcU...............................................................................................................................................................   1    1    JCK-3
```
